# Characterizing virus-induced gene silencing at the cellular level with in situ multimodal imaging

**DOI:** 10.1186/s13007-018-0306-7

**Published:** 2018-05-25

**Authors:** Sadie J. Burkhow, Nicole M. Stephens, Yu Mei, Maria Emilia Dueñas, Daniel J. Freppon, Geng Ding, Shea C. Smith, Young-Jin Lee, Basil J. Nikolau, Steven A. Whitham, Emily A. Smith

**Affiliations:** 1 The Ames Laboratory, U.S. Department of Energy, Iowa State University, Ames, IA 50011-3111 USA; 2 Department of Chemistry, Iowa State University, Ames, IA 50011-3111 USA; 3 Department of Plant Pathology and Microbiology, Iowa State University, Ames, IA 50011 USA; 4 Department of Biochemistry Biophysics, and Molecular Biology, Center for Metabolic Biology, Iowa State University, Ames, IA 50011 USA; 5 Engineering Research Center for Biorenewable Chemicals, Iowa State University, Ames, IA 50011 USA

**Keywords:** RNA silencing, *Foxtail mosaic virus*, *Phytoene desaturase*, Subcellular Raman imaging, Mass spectrometry imaging, Whole-plant analysis, Biochemical characterization, Mosaic spatial pattern, Carotenoids

## Abstract

**Background:**

Reverse genetic strategies, such as virus-induced gene silencing, are powerful techniques to study gene function. Currently, there are few tools to study the spatial dependence of the consequences of gene silencing at the cellular level.

**Results:**

We report the use of multimodal Raman and mass spectrometry imaging to study the cellular-level biochemical changes that occur from silencing the *phytoene desaturase* (*pds*) gene using a *Foxtail mosaic virus* (FoMV) vector in maize leaves. The multimodal imaging method allows the localized carotenoid distribution to be measured and reveals differences lost in the spatial average when analyzing a carotenoid extraction of the whole leaf. The nature of the Raman and mass spectrometry signals are complementary: silencing *pds* reduces the downstream carotenoid Raman signal and increases the phytoene mass spectrometry signal.

**Conclusions:**

Both Raman and mass spectrometry imaging show that the biochemical changes from FoMV-*pds* silencing occur with a mosaic spatial pattern at the cellular level, and the Raman images show carotenoid expression was reduced at discrete locations but not eliminated. The data indicate the multimodal imaging method has great utility to study the biochemical changes that result from gene silencing at the cellular spatial level of expression in many plant tissues including the stem and leaf. Our demonstrated method is the first to spatially characterize the biochemical changes as a result of VIGS at the cellular level using commonly available instrumentation.

**Electronic supplementary material:**

The online version of this article (10.1186/s13007-018-0306-7) contains supplementary material, which is available to authorized users.

## Background

Reverse genetics techniques, such as RNA silencing, have been widely used over the past 20 years to generate loss-of function phenotypes that provide insight into the functions of silenced genes. Virus-induced gene silencing (VIGS) is a method of RNA silencing that takes advantage of the plant’s natural antiviral defense mechanisms. VIGS requires a modified viral vector that carries RNA or DNA fragments corresponding to the plant target gene(s). The recombinant virus replicates and moves systemically throughout the plant. Meanwhile the antiviral RNA silencing system is activated against the viral genetic template, which also encompasses the target plant gene fragment, resulting in the silencing of the target gene. The use of VIGS technologies addresses the need for rapid and potentially high-throughput methods for testing gene functions in a wide variety of monocot and dicot plant species [1–11].

**Fig. 1:**
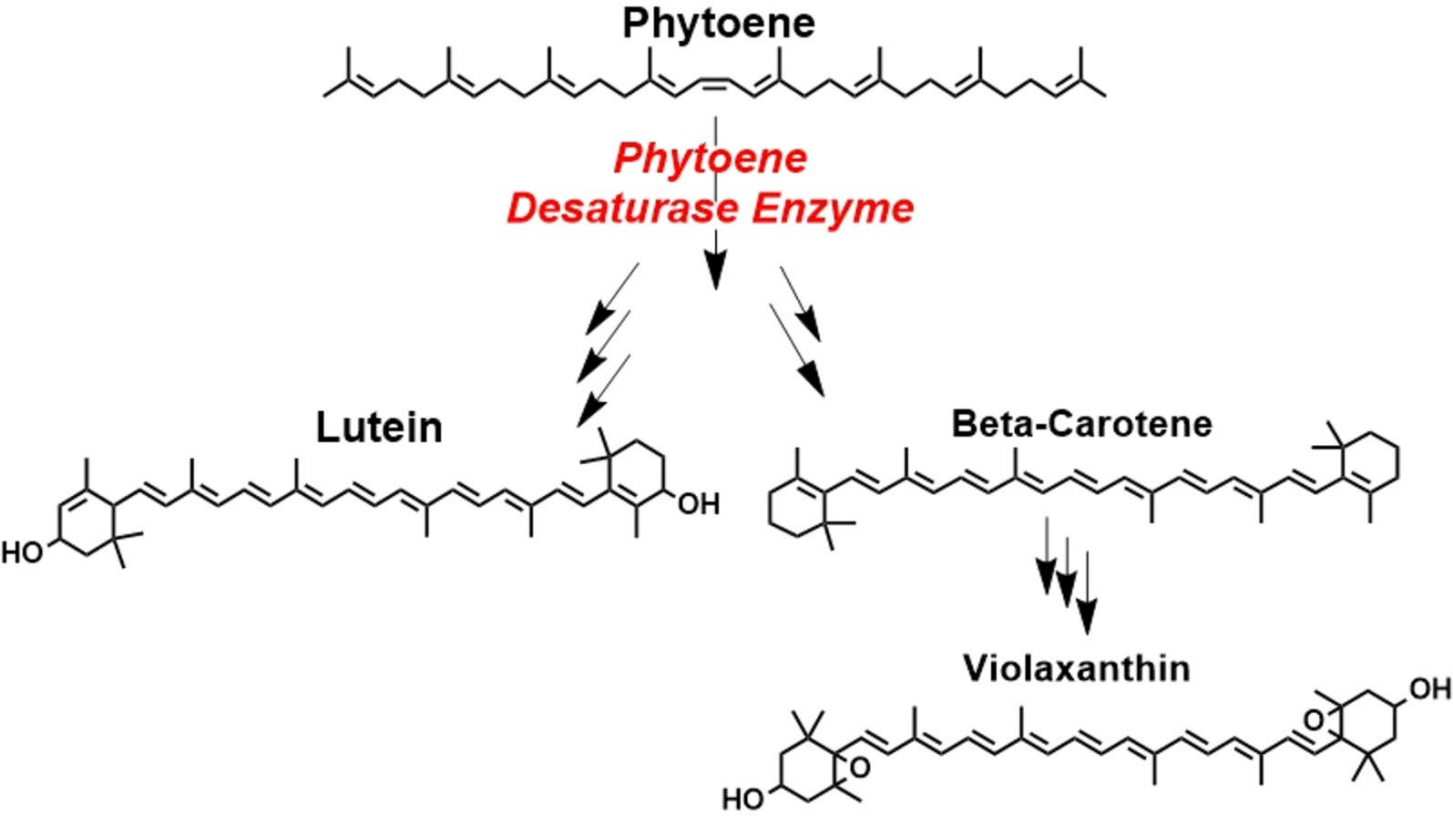
Simplified carotenoid biosynthesis pathway in higher plants: phytoene (absorption and emission λ, 291, 360 nm, respectively), β-carotene (absorption and emission λ, 465, 542 nm, respectively), lutein (absorption and emission λ, 456, 525 nm, respectively), and violaxanthin (absorption and emission λ reported by Gruszecki et al. [57], 410, 555 nm, respectively)

*Foxtail mosaic virus* (FoMV), belonging to the *Potexvirus* genus, was recently developed as a VIGS vector for use in maize and other important monocot crop species such as wheat [1, 2]. The genome organization of FoMV and other potexviruses consists of five major open reading frames (ORFs) encoding: RNA polymerase (ORF1), the triple gene block (ORF2-4), and the coat protein (ORF5) [10, 12], all of which are essential for virus survival and function. In addition, FoMV encodes a unique 5A protein that is not essential for replication or viral infection [12]. Mei et al. [1] developed a DNA-based full-length FoMV VIGS vector by inserting a cloning site after the coat protein. This FoMV vector was used to silence *phytoene desaturase* (*pds*) and other genes in sweet corn and the B73 inbred line of yellow dent corn. Silencing *pds* in tobacco leaves has been shown to produce an easily observed variegated white phenotype [11]. The *pds* enzyme along with other desaturases and isomerases convert the colorless phytoene molecule to downstream carotenoids (Fig. 1) [13–18]. These downstream carotenoids have multiple conjugated double bonds that lead to the absorption of light in the visible region (~ 390–700 nm). Zhang et al. [19] utilized a *Bean pod mottle virus* VIGS vector to silence *pds* within soybean leaves. They tagged this VIGS vector with green fluorescent protein (GFP), and confirmed via fluorescence that the vector was spatially correlated to the visual mosaic phenotype produced from silencing *pds* [19]. Juvale et al. [20] performed a similar experiment with transgenic soybeans that constitutively expressed a GFP transgene in all tissues to measure GFP VIGS from a *Bean pod mottle virus* vector [20]. They determined that the GFP transgene was uniformly silenced and suggested the differences between their observation and those reported by Zhang et al. [19] may result from silencing a GFP transgene versus endogenous *pds*. While a fluorescent tag can be used to localize the VIGS vector, the fluorescence signal does not reveal downstream biochemical effects occurring from gene silencing.

Raman and mass spectrometry (MS) imaging are complementary analysis techniques in regard to the nature of the signal, the kind of information measured, and the spatial resolution. Raman spectroscopy provides a fingerprint of functional groups within a molecule and is an ideal tool for carotenoid characterization. Raman scattering results when there is a change in photon energy as a result of exciting vibrations in chemical bonds. Resonance Raman spectroscopy (and pre-resonance Raman spectroscopy) occurs when the laser excitation wavelength falls within the range of wavelengths absorbed by a molecule and the result is an enhanced Raman scattering intensity. Raman imaging is a non-destructive technique that can be performed on whole tissue (such as fruit, leaves, stems, roots) or sections of these tissues and provides spatially-localized chemical information for a variety of compounds. The spatial distribution, relative content, and accumulation of carotenoids within plant tissues can be measured by plotting the area of a Raman peak to generate a Raman image [21–28]. These compounds, however, generally need to be abundant to be measured with Raman techniques. MS imaging is suitable to measure low abundance compounds with a greater chemical selectivity, and has become a valuable analytical tool for analyzing the spatial distribution of a wide range of compounds directly on or within plant tissues [29–32]. Matrix-assisted laser desorption/ionization (MALDI) imaging provides high sensitivity, and chemical versatility. Nanoparticles are efficient matrices for small molecule analysis using MALDI-MS imaging due to their low or negligible matrix background, homogeneous application, and high laser absorption [33]. In particular, silver nanoparticles have been commonly used for analyte cationization of various molecules, such as cholesterol, fatty acids, and other olefin-containing molecules [34–36].

Herein, a combined Raman and MS imaging approach is presented for the measurement of the cellular spatial dependence of *pds* silencing using the FoMV vector developed by Mei et al. [1] in the leaves of the maize sweet corn variety Golden × Bantam. The goal of this work is to gain a cellular level understanding of a VIGS phenotype using the downstream biochemical changes in carotenoid expression that occur from silencing *pds*. We present a useful methodology suitable to study the spatial dependence of gene silencing at the cellular level using any VIGS virus targeting genes that produce a unique biochemical signature within leaf and stem tissues.

## Methods

### FoMV-*pds* silenced, FoMV, and non-inoculated sweet corn line golden × bantam plants

Plants were grown and inoculated as described by Mei et al. [1]. Plants were grown in a 20–22 °C greenhouse with a 16-h photoperiod. A Biolistic PDS-1000/He system was utilized to inoculate 1-week-old plants by particle bombardment with FoMV infectious clones (Bio-Rad Laboratories). The biolistic inoculation used 1 µm gold particles coated with 1 µg of FoMV plasmid DNA at 1100 p.s.i. to rupture disks from a distance of 6 cm. Plants were placed in the dark for 12 h before and after bombardment with the FoMV infectious clones. The infected leaves were ground to sap with 50 mM phosphate buffer (pH 7) and then rubbed onto plants with 600-mesh Carborundum at the two-leaf stage. The rub-inoculated plants were considered the “FoMV-*pds* silenced” plants. The “FoMV” plants were rub-inoculated with the FoMV vector without the sequence encoding the *pds* gene. “Non-Inoculated” plants were not inoculated with the FoMV vector. The following titles FoMV-*pds*, FoMV, and non-inoculated will be used throughout this work to describe the three plant types. Plants were approximately 5–6 weeks old or at the 5–9 leaf stage when measurements were completed. For all imaging experiments, the total leaf length was measured and the position representing half of the total leaf length was used for measurements. For full information about the VIGS system see Mei et al. [1]. Quantitative reverse transcription polymerase chain reaction (qRT-PCR) was used to determine the effectiveness within FoMV-*pds* silenced leaves. In leaves where the variegated phenotype was observed, there was a significant reduction (13.5–27.6%) of *pds* expression when compared to the FoMV or non-inoculated leaves [1]. The VIGS methodology is also described in Mei et al. [37].

### Transverse cross sections of maize leaves

A Leica CTI cryostat was utilized to section the leaves to 45 µm thickness. Leaves 5 and 6 were manually cut with a scalpel into 5–8 cm samples at the half-leaf-length position. The FoMV-*pds* silenced leaves were sectioned at the highest abundance of variegated white areas at the half-leaf-length position. The cut samples were flash frozen in liquid nitrogen for 30 s. The fixed tissues were placed vertically in the cryostat base molds which were filled halfway with distilled water. The samples were left in the cryostat set at − 23 °C until the water was frozen. Sectioned samples were placed onto a microscope slide with a drop of distilled water, sealed with a glass coverslip and clear nail polish. Samples were stored in the dark and analyzed within 12 h of being sectioned.

### Fractured maize leaves

Leaves were prepared as outlined previously by Klein et al. [38]. Leaves 5 and 6 were manually cut with a scalpel into 3–5 cm samples at the half-leaf-length position. The cut samples were placed on packing tape and vacuum dried. Once dried, the packing tape was folded over, passed through a rolling mill, and the two halves of the leaf were separated by pulling the tape apart.

### Mass spectrometry (MS) imaging

The fractured maize leaves were subject to matrix deposition by sputter coating (108 Auto Sputter Coater, Ted Pella INC, Redding, CA, USA) silver at 40 mA for 10 s. MS imaging data were collected using a MALDI-linear ion trap (LIT)-Orbitrap mass spectrometer (MALDI-LTQ-Orbitrap Discovery; Thermo Finnigan, San Jose, CA, USA). The instrument was modified to incorporate an external 355-nm frequency tripled Nd: YAG laser (UVFQ; Elforlight, Daventry, UK). TunePlus and XCalibur (ThermoFisher Scientific) were used to define imaging parameters and to acquire data, respectively. Mass spectra were acquired with 10 laser shots per spectrum in positive ion mode using an Orbitrap mass analyzer (resolution 30,000 at m/z 400) for an *m/z* scan range of 100–1000 and using a 20 µm raster step size. MS images were generated using ImageQuest (ThermoFisher Scientific) with a mass window of ± 0.003 Da. The imaged peak is a silver adduct, [M + ^107^Ag]^+^ of phytoene. MS/MS analysis was performed on a different region of the fractured leaf tissue to the one used for MS imaging. The ion-trap analyzer was used for MS/MS of selected ions with a mass window of 1.5 Da and a normalized collision energy of 35 (arbitrary units). No downstream carotenoids were detected by MS imaging for any of the maize leaves using these experimental conditions suitable for detecting phytoene.

### Raman imaging

Raman measurements were performed using a commercially available XploRA Plus Raman confocal upright microscope with a Synapse EMCCD camera (HORIBA Scientific, Edison, New Jersey). An Olympus objective (20 × magnification, 0.4 numerical aperture) was used to collect images in the epi-direction with a 1200 grooves/millimeter grating and a 1450 cm^−1^ center wavelength, and a 100 µm confocal pinhole. A 532-nm solid-state diode laser produced an 800 W/cm^2^ laser irradiance, unless otherwise noted. A 75 × 50 mm XYZ translational stage and a step size of 3 µm was utilized for all Raman images. The size (and acquisition time) for the in situ whole leaf, leaf transverse cross sections, and fractured leaf Raman images were 50 × 100 µm (15 s), 120 × 238 µm (5 s), and 50 × 50 µm (15 s), respectively. Two measurements per leaf and two separate leaves were measured for each plant type (i.e., FoMV-*pds*, FoMV, and non-inoculated). For the in situ whole leaf and leaf transverse cross sections. For the fractured leaf images, 2 areas on each side of the fractured leaf were collected for each plant type. White light optical images of the same area corresponding to the Raman images were collected using the same instrument. ImageJ was used to analyze optical images.

Igor Pro 6.36 (WaveMetrics, Inc., Lake Oswego, OR) scientific analysis and graphing software was used to process the Raman spectra. A Gaussian function with a constant baseline was used to batch fit and extract the ~ 1520 cm^−1^ peak amplitudes and maxima. In order to generate Raman images, an automated method applied two criteria to the output of the resulting Gaussian fit functions: the peak maximum was between 1515 and 1530 cm^−1^, and the peak intensity had to be larger than three times the standard deviation of the noise. The noise was quantified within the region of 500–600 cm^−1^ where no spectral peaks are located. If any of the criteria were not met, the corresponding pixel was assigned null (a gray pixel) within the Raman image. The Raman images were plotted using a custom MATLAB 2016b script. All reported uncertainties represent one standard deviation.

Histograms of the ~ 1520 cm^−1^ peak maximum were compiled for all spectra in four Raman images (in situ whole leaf and transverse leaf cross sections) or two images (fractured leaf). The bin width of the histogram was 1 cm^−1^, and the histogram was fit to a Gaussian function to obtain the reported distribution mean.

### Standard and supplement Raman measurements

β-carotene (Sigma-Aldrich) and phytoene (Toronto Research Chemicals) standards were diluted to 0.01 mg/mL in chloroform for absorbance and fluorescence measurements. Absorbance measurements were performed on an Agilent 8453 UV–visible spectrophotometer. Fluorescence measurements were carried out on an Agilent Cary Eclipse spectrophotometer. The excitation wavelength for fluorescence measurements was 291 nm for phytoene and 464 nm for β-carotene. For Raman measurements, the pure standards were diluted to 0.25 mg/mL in chloroform and 3 µL were drop casted (solvent evaporated) onto a clean (soaked in isopropanol and dried under a nitrogen flow) glass microscope cover slip. Raman spectra were collected with a 3 s acquisition, 2 accumulations and 1.66 W/cm^2^ laser irradiance.

Lutein (Nature’s Bounty©), zeaxanthin (Swanson Ultra©), lycopene (Spring Valley©), and β-carotene (Nature’s Bounty©) liquid capsule dietary supplements were purchased from local retailers. Raman measurements required the contents of an individual capsule to be emptied onto a clean microscope slide. To collect the Raman spectrum of the supplement mixture, an equal volume of all the supplements was mixed together. The acquisition parameters were: 10 s acquisition, 2 accumulations and 1.66 W/cm^2^ laser irradiance.

## Results

### Carotenoid characterization by Raman spectroscopy

**Fig. 2:**
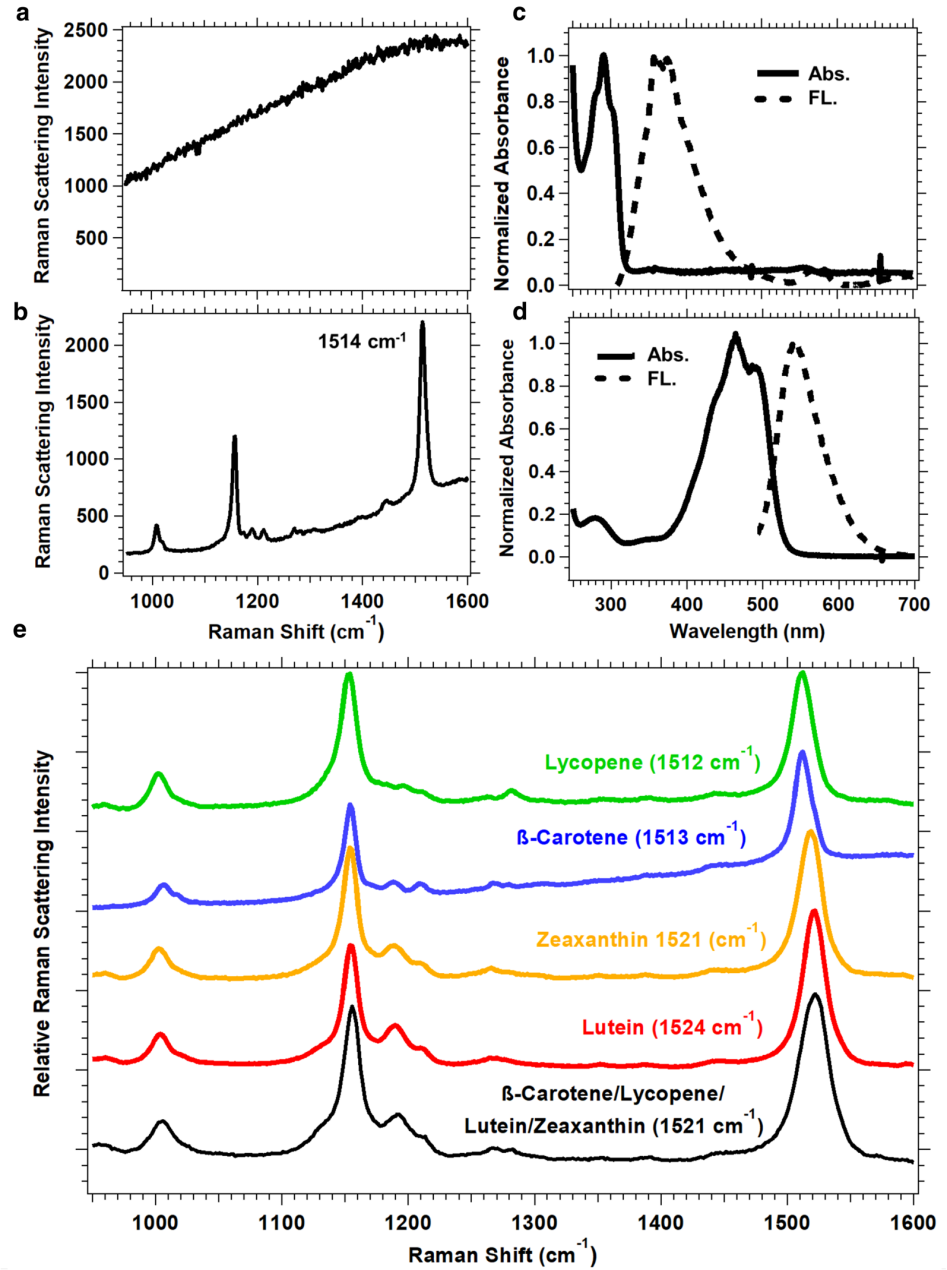
Spectra of selected carotenoid** a**–**d** standards and **e** supplements.** a** Raman, **c** absorption and fluorescence spectra of phytoene. **b** Raman,** d** absorption and fluorescence spectra of β-carotene. The phytoene absorption maximum is ~ 291 nm, which is not resonantly enhanced with a 532 nm laser, leading to a lack of peaks in the Raman spectrum. **e** Raman spectra of lycopene, β-carotene, zeaxanthin, lutein, and a mixture of the supplements with corresponding peak maxima

Raman spectra of phytoene and β-carotene standards were measured to understand the Raman signal of these compounds and ultimately to enable the interpretation of the Raman images obtained from the maize leaves. The Raman spectrum of phytoene, when collected with a 532 nm laser, showed only a broad background and no Raman peaks (Fig. 2a), whereas the Raman spectrum of β-carotene showed peaks that are characteristic of carotenoids (Fig. 2b). There were three major vibrational modes (ν) in the Raman spectrum of carotenoids [22, 26, 39–42]. The most intense ν_1_ band corresponded to the in-phase stretching vibrations of C=C bonds, and occurred between 1512 and 1524 cm^−1^. The ν_2_ band corresponded to the stretching vibrations of C–C bonds, and occurred at approximately 1150 cm^−1^. The lowest intensity ν_3_ band corresponded to the C-H stretching modes, and occurred at approximately 1000 cm^−1^. The absorption spectra revealed the reason for the differences in the Raman spectra measured for phytoene and β-carotene. Phytoene does not absorb 532 nm light (Fig. 2c) and thus does not exhibit resonant enhancement of the Raman signal, whereas β-carotene does absorb 532 nm light (Fig. 2d) and will exhibit an enhanced Raman signal, as will any carotenoid that absorbs the excitation wavelength.

Carotenoid supplements were utilized to determine how the Raman peaks were affected by their varying chemical structure (Fig. 2e). All the analyzed carotenoids absorbed 532 nm light to varying extents (Additional file 1: Fig. S1) and produced a strong Raman signal under these experimental conditions. There were few differences in the Raman peaks below 1450 cm^−1^. There were, however, differences in the ~ 1520 cm^−1^ peak maximum, and these may allow changes in carotenoid mixtures to be identified by Raman spectroscopy. The ~ 1520 cm^−1^ peak maximum depended on the degree of conjugation as well as the substituents on the tetraterpenoid backbone of the carotenoid. Lycopene and β-carotene possess 11 conjugated double bonds and no hydroxyl substituents and yielded a peak maximum at 1512 and 1513 cm^−1^ (Fig. 2e). The chemical structure of zeaxanthin and lutein have two hydroxyl substituents and the peak maximum was observed at 1521 and 1524 cm^−1^ (Fig. 2e). The spectral resolution of the Raman instrument determined whether the peak maxima can be distinguished. The instrument used to collect the Raman images in this work had a 5 cm^−1^ spectral resolution based on a spectral calibration with a Ne lamp. Thus, within the same spectrum β-carotene or lycopene peak maxima could be distinguished from lutein or zeaxanthin. A mixture of all four supplements had a peak maximum at 1521 cm^−1^ with a Gaussian peak shape and no evidence of spectrally overlapping peaks (Fig. 2e). For carotenoid mixtures, the peak maximum is expected to depend on the chemical composition and concentration of the constituents.

### Cellular spatial distribution of carotenoids: whole leaf measurements

**Fig. 3:**
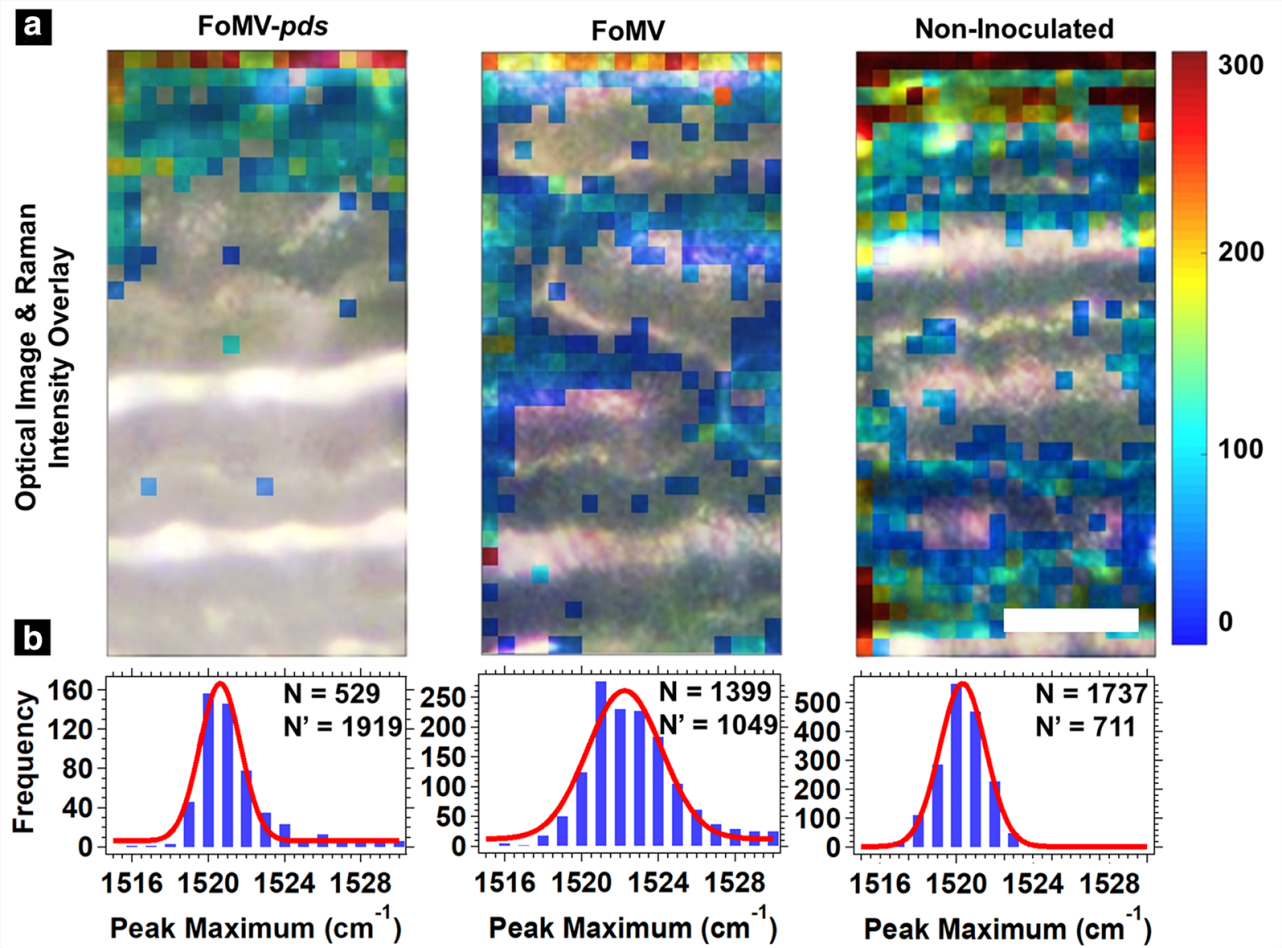
**a** Merged optical and ~ 1520 cm^−1^ Raman images of in situ whole maize leaves. From left to right the images correspond to FoMV-*pds* silenced, FoMV, and non-inoculated leaves. The color scale at right represents the Raman scattering intensity and is the same for all images. The scale bar is 25 µm and is the same for all images. Additional in situ whole leaf Raman images are shown in Additional file 1: Fig. S2, and optical images of an expanded area around where the Raman images were collected are shown in Additional file 1: Fig. S3.** b** Histograms of the ~ 1520 cm^−1^ peak maximum were generated from each pixel in the Raman images shown in **a** and Additional file 1: Fig. S2. The histograms were fit to a Gaussian function (red solid line). N is the number of pixels that were positive for carotenoids and N′ is the number of pixels that were null (not included in the histogram)

Overlays of the optical (white light illumination) and Raman images collected in situ on whole leaves are shown in Fig. 3a and Additional file 1: Fig. S2. The intensity of the ~ 1520 cm^−1^ carotenoid peak was the largest and used to generate the Raman images to ensure the best sensitivity. The cell types visualized in the optical images were epidermal and guard cells. The white, primarily horizontal, stripes observed in all the optical images were only visible when the light was illuminating the leaf on the same side as the detector (epi-illumination) as opposed to when the illumination and detector were on opposite sides (trans-illumination) (Additional file 1: Fig. S4). This indicated the striped features were generated from light reflection or scattering from the exterior (cuticle) layer of the leaf. A representative camera image of the FoMV-*pds*, FoMV, and non-inoculated leaves with *pds* silenced areas outlined within the FoMV-*pds* plant type is shown within Additional file 1: Fig. S5). Within the FoMV-*pds* silenced leaf, the carotenoid signal was not measured in most of the pixels at the bottom of the image where the FoMV mosaic pattern was visually observed (Fig. 3a). (Optical images of the areas adjacent to where the Raman images were collected are shown in Additional file 1: Fig. S3). This indicated a reduction in carotenoid expression within these measured areas. The few pixels that showed a carotenoid signal in the variegated white phenotype area were located where the scattering/reflection of light at the leaf surface was the lowest and may represent areas where the excitation light penetrates farther into the plant tissue. For the FoMV and non-inoculated plants (Fig. 3a), the carotenoid signals were distributed more evenly throughout the Raman image. The peak maximum histograms for the FoMV-*pds* silenced, FoMV, and non-inoculated leaves (Fig. 3b) had a mean ranging from 1521 to 1522 cm^−1^, suggesting when carotenoids were present there was a similar carotenoid composition in all plant types.

### Cellular spatial distribution of carotenoids: transverse leaf cross section measurements

**Fig. 4:**
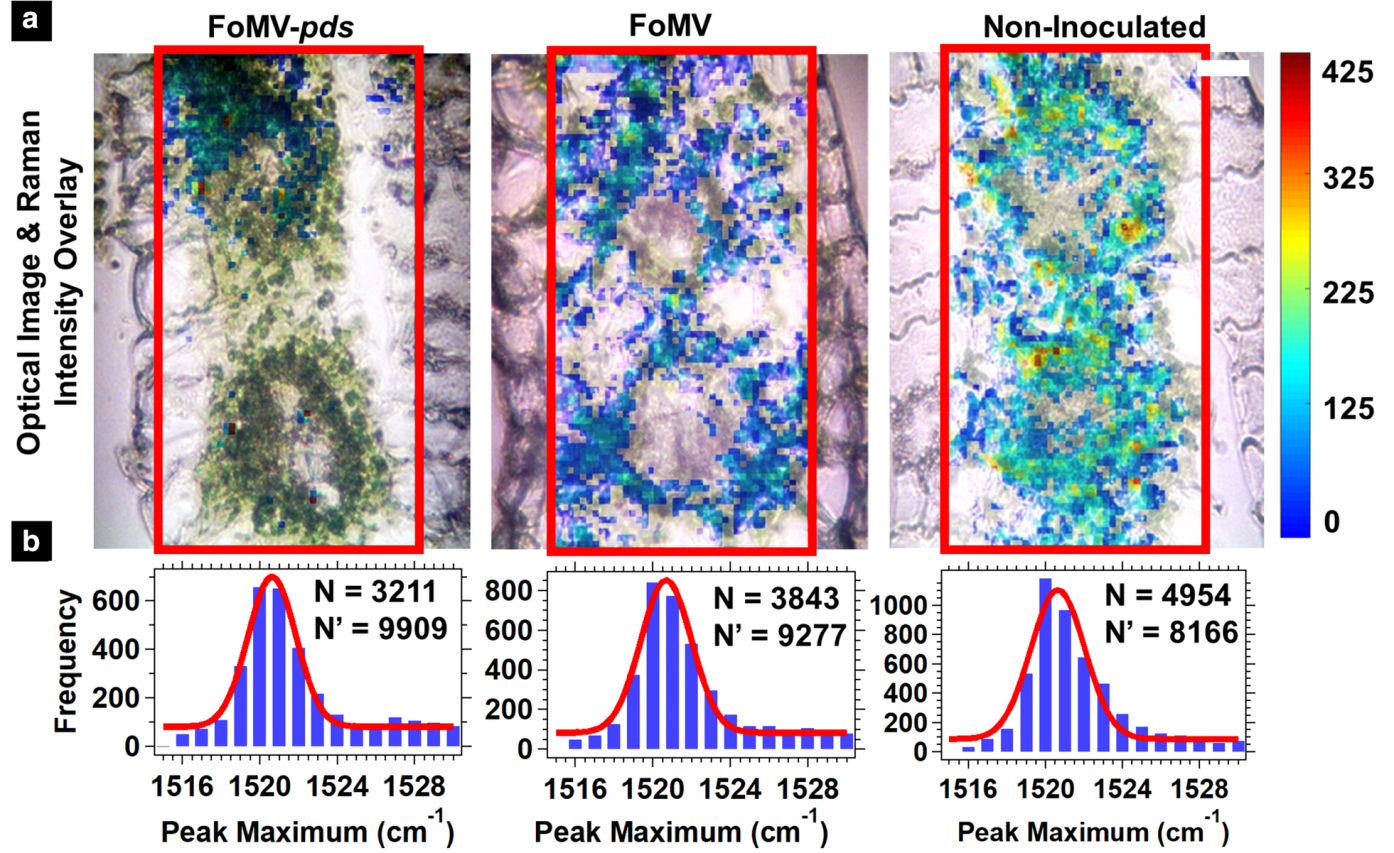
**a** Merged optical and ~ 1520 cm^−1^ Raman images (outlined in red) of maize leaf transverse cross sections. From left to right the images correspond to FoMV-*pds* silenced, FoMV, and non-inoculated leaves. The color scale represents the Raman scattering intensity as shown in the scale at right. The scale bar is 25 µm. Additional transverse cross section Raman images are shown in Additional file 1: Fig. S6. **b** Histograms of the ~ 1520 cm^−1^ peak maximum were generated from each pixel in the Raman images shown in **a** and Additional file 1: Fig. S6. The histograms were fit to a Gaussian function (red solid line). N is the number of pixels that were positive for carotenoids and N′ is the number of pixels that were null (not included in the histogram)

As mentioned above, the excitation laser may have probed through different tissue depths at different locations on the whole leaf, and the in situ whole leaf measurements were not ideal for obtaining information about internal cellular structures including the mesophyll and vascular bundles. Raman images of transverse cross sections of the leaf revealed the internal cellular structures (Fig. 4). Each image showed at least two vascular bundles with the surrounding mesophyll, and epidermal cells present on the left and right of the vascular bundles (Fig. 4a). The FoMV and non-inoculated optical and Raman images of the transverse cross sections showed a correlation between chloroplast location and carotenoid signal. The correlation of chloroplast location and carotenoid signal were in agreement with the location of carotenoid biosynthesis [43]. The overall carotenoid signal from replicates of the same plant type (FoMV-*pds*, FoMV, and non-inoculated) (Additional file 1: Fig. S6), showed extreme variability, for this reason no conclusions about differences in the spatial location of the carotenoid signal among the three plant types were made from data collected with this sample preparation method. The ~ 1520 cm^−1^ peak maximum histograms generated from the transverse cross section data (Fig. 4b) were consistent with the values obtained for the in situ whole leaf measurements (Fig. 3b) with a distribution maximum between 1521 and 1522 cm^−1^, indicating a similar carotenoid composition is measured in both sample preparation methods.

### Complementary Raman and mass spectrometry imaging of fractured maize leaves

**Fig. 5:**
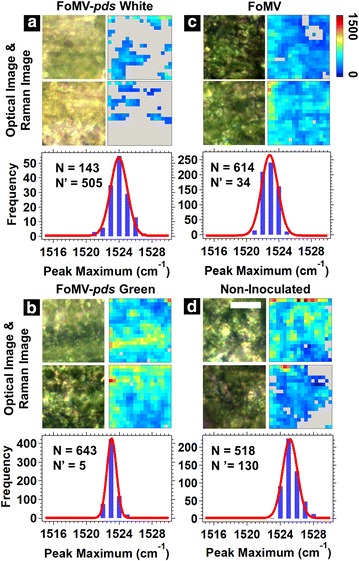
**a**–**d** Optical images, ~ 1520 cm^−1^ Raman images of fractured maize leaves, and histograms of the ~ 1520 cm^−1^ peak location generated from each pixel within the Raman images. The fracturing process splits the leaf into two halves lengthwise. For each panel, the top optical and Raman image corresponds to one side of the fractured leaf, and the bottom optical and Raman image corresponds to the other side of the fractured leaf. Two areas at the half-leaf-length position were analyzed for the FoMV-*pds* leaf: **a** variegated white area and **b** a green area. The scale bar is 25 µm. The histograms were fit to a Gaussian function (red solid line). **c**, **d** represent the area measured for the FoMV and non-inoculated fractured leaves, respectively. N is the number of pixels that were positive for carotenoids and N′ is the number of pixels that were null (colored gray and were not included in the histogram)

Fracturing the leaves provided a way to consistently correlate the Raman signal from internal leaf structures and the visible phenotype observed from the exterior of the leaf, although some cellular structures may have been altered from the fracturing process (Fig. 5). Raman images collected on the variegated white areas of the FoMV-*pds* silenced fractured leaves showed low, but not zero, levels of carotenoid Raman signal (Fig. 5a); whereas the green areas (Fig. 5b) showed carotenoid signals consistent with the FoMV (Fig. 5c) and non-inoculated (Fig. 5d) fractured leaves with a moderately uniform carotenoid signal. The ~ 1520 cm^−1^ peak maximum histogram mean ranged from 1523 to 1525 cm^−1^ for the FoMV-*pds* silenced, FoMV, and non-inoculated fractured leaves.

**Fig. 6:**
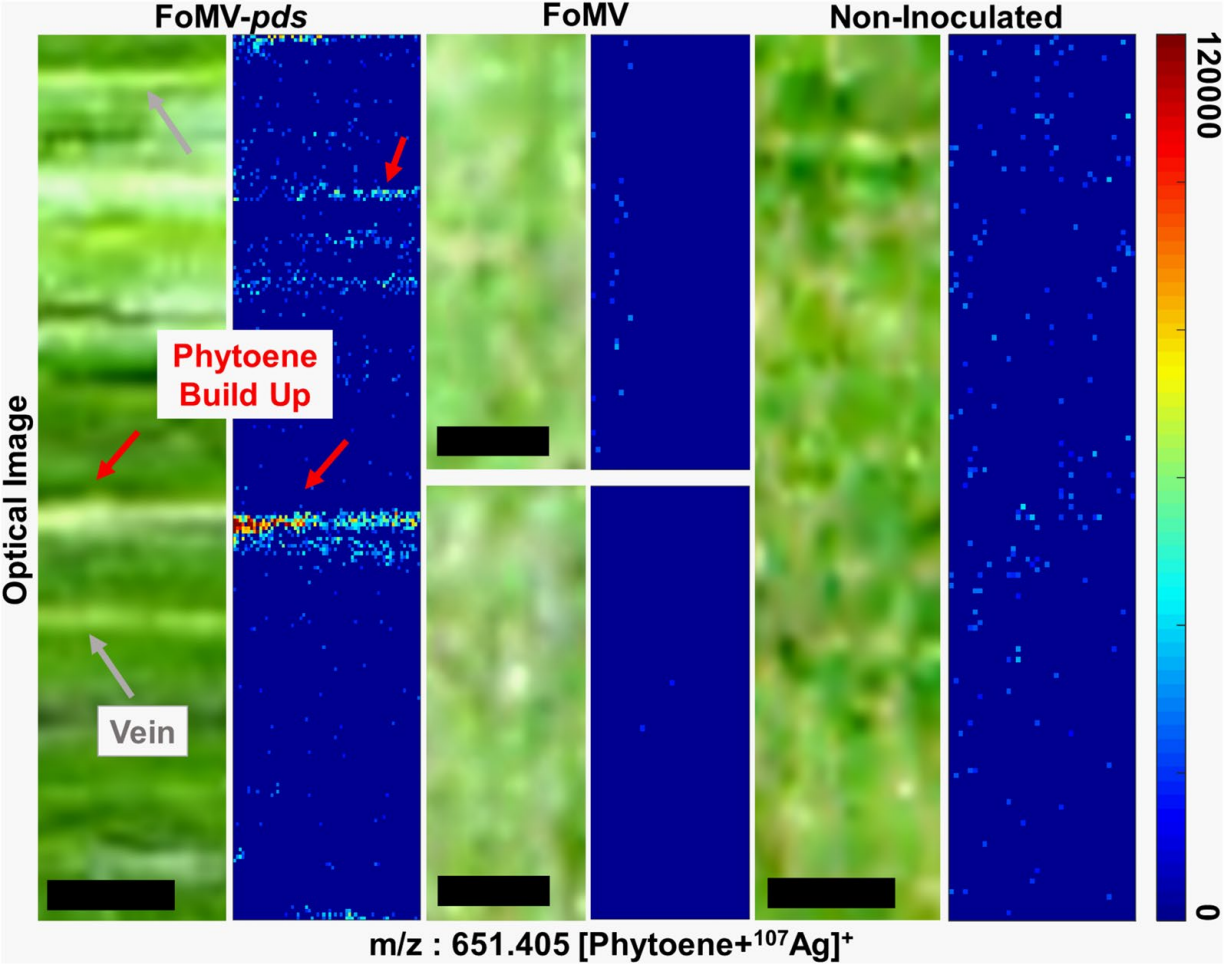
Mass spectrometry (MS) images of fractured FoMV-*pds* silenced, FoMV and non-inoculated maize leaves. The images are generated using the phytoene mass-to-charge (m/z) ratio 651.405, which is a silver adduct [Phytoene + ^107^Ag]^+^. The ion intensity is shown using the color scale shown on the right. The leaf vein(s) were identifiable (gray arrows) because they were present along the entire length of the leaf. The FoMV-*pds* silenced variegated white areas (red arrows) however were not present along the entire length of the leaf. The resolution of the camera collecting the optical images on the MSI instrument is low, however an additional optical image collected prior to imaging is provided in Additional file 1: Fig. S8. Scale bar is 500 µm for all images

Phytoene did not produce an appreciable Raman signal with the experimental conditions used for this study, therefore MS imaging was used to detect phytoene and confirm the spatial localization within the fractured leaves. A sputter coated silver matrix was used for detection. The *m/z* assignment (*m/z* 651.405, [M + ^107^Ag]^+^) was based on accurate mass measurement, and confirmed by MS/MS [44] (Additional file 1: Fig. S7). Low phytoene signal was present in the MS image of the FoMV and non-inoculated fractured leaf, indicating there was no appreciable buildup of phytoene (Fig. 6). On the contrary, there was a specific localization of phytoene signal within the FoMV-*pds* silenced fractured leaf originating from the accumulation of phytoene.

High performance liquid chromatography (HPLC) was performed on the leaf samples to quantify the concentration of selected carotenoids (Additional file 1: Table S1). The concentration of phytoene extracted from the FoMV-*pds* leaf and analyzed by HPLC was 0.4 ± 0.2 µg/g of fresh weight (see Additional file 1 for extraction method). Phytoene was not detected by HPLC in the extract of the non-inoculated leaves and was measured at a very low concentration 0.011 ± 0.005 µg/g of fresh weight within two of the replicates for the FoMV leaf. (The HPLC limit of detection for phytoene was 0.003 µg/g of fresh weight.) The quantification performed with the extract was consistent with the MS images that showed a very low phytoene signal across the FoMV and non-inoculated leaf. The downstream carotenoids (Fig. 1) measured in the non-inoculated leaf were: lutein (61 ± 3 µg/g), α and β-carotene (22 ± 1 µg/g), violaxanthin/neoxanthin (10 ± 2 µg/g), and zeaxanthin (7 ± 5 µg/g). Comparing the FoMV leaf to the non-inoculated leaf, the only statistically significant difference (*p* < 0.05) was a lower concentration of α and β-carotene within the FoMV leaf. Comparing the FoMV-*pds* leaf to the non-inoculated leaf there were no statistically significant differences in the downstream carotenoid concentrations. This observation justifies a need for chemical imaging to provide biochemical information at specific spatially-correlated locations to measure changes in the local carotenoid expression that were lost in the spatially-averaged signal that is obtained from the extract analysis.

## Discussion

The most common method used to analyze the effectiveness of VIGS is qRT-PCR [1, 9]. The mRNA transcript levels are generally measured using a large amount of tissue (i.e., spatially averaged) and are reported as a percentage change relative to non-inoculated tissue. This technique is not able to reveal downstream biochemical effects occuring as a result of VIGS at the cellular level. While visual inspection of the plant tissue can reveal the macroscopic variegation that results from *pds* silencing, such a simple analysis cannot reveal details at the cellular level, provide information about specific biochemical compounds, nor easily differentiate varying low levels of response. FoMV-*pds* suppresses expression of the mRNA transcripts encoding the *pds* enzyme and this is expected to result in the accumulation of the colorless phytoene in leaves [11, 45]. The accumulation of phytoene cannot be detected by a visible phenotype. The analysis of the extracted carotenoids demonstrates an increase in phytoene within the FoMV-*pds* silenced leaf. MS imaging revealed an increased phytoene signal that was spatially localized within the variegated areas of the fractured leaves of the FoMV-*pds* silenced leaves (Fig. 6). This agrees with the reduced carotenoid Raman signal within the same area (Fig. 5). The FoMV and non-inoculated fractured leaf MS images display a low non-localized phytoene signal. Even though *pds* silencing does not result in the complete elimination of the mRNA expression within FoMV-*pds* tissues [1], the multimodal imaging technique still measures the biochemical changes in carotenoid expression that occurred from the achieved level of gene silencing. The measurable changes are expected to be easier to detect by the outlined imaging approach when the silencing levels are higher.

The carotenoid extract shows no statistically significant differences in any of the carotenoids measured by HPLC when comparing the non-inoculated and FoMV-*pds* leaves. This is not surprising when considering the mosaic pattern of infection and *pds* silencing only affects a small area of the overall leaf. It is difficult to quantify small differences in the bulk of the extracted components. Imaging approaches, on the other hand, allow for a localized distribution to be measured and reveal spatially-correlated differences lost in the average when analyzing an extraction. The percentage of pixels that have a detectable carotenoid signal within the Raman images for the FoMV-*pds* silenced in situ whole leaves is 22 ± 5% (n = 4) in the areas measured. The percentage of pixels that have a detectable carotenoid signal for FoMV and non-inoculated in situ whole leaves are 57 ± 9% and 70 ± 10%, respectively. The fractured leaf images reveal similar results. The percentage of pixels that have a detectable carotenoid signal for FoMV-*pds* silenced, FoMV, and non-inoculated fractured leaves are 22 ± 2% (n = 2), 99.1 ± 0.3%, 80 ± 8%, respectively. Within the region that produces a visible mosaic phenotype, the carotenoid expression is reduced but not eliminated and is most evident in the fractured leaf images.

The most abundant downstream carotenoids reported in the literature for the leaves of maize and other monocots are lutein, β-carotene, and violaxanthin [46–50]. The extracted carotenoids measured by HPLC are (in order of abundance): lutein, α and β-carotene, violaxanthin/neoxanthin, and zeaxanthin. Other carotenoids may also be present as a comprehensive HPLC analysis has not been performed. The histograms of the ~ 1520 cm^−1^ Raman peak maximum provide information about the nature of the carotenoids present. The 1521–1522 cm^−1^ peak maximum measured for the in situ whole leaf and transverse cross section is consistent with a mixture consisting of primarily lutein and β-carotene. The fractured leaves have a 1–5 cm^−1^ higher mean peak maximum compared to the in situ whole leaf and transverse cross section measurements, which is not likely the result of biologically relevant differences in chemical compositions since the same samples were analyzed for the three leaf preparation methods. Carotenoids are susceptible to oxidation, especially if the plant is under stressors including excess light or dehydration [51–55]. The preparation of a fractured leaf requires dehydration, unlike the other two sample preparation methods, and oxidation of the carotenoids upon fracturing the leaf may explain the shift in the peak maximum to higher wavenumbers.

Raman and MS imaging are complementary imaging approaches in many aspects: (i) non-destructive versus destructive sampling; (ii) subcellular resolution of ~ 1 µm versus a cellular resolution of ~ 10 µm; (iii) knowledge of functional groups present versus the mass of the molecules present; and (iv) molecular sensitivity to resonance with the excitation laser for Raman imaging versus a high ionization efficiency with MS imaging. Whereas the differential sensitivity of MS imaging enables the imaging of phytoene, the precursor to downstream carotenoids, Raman imaging provides high selectivity for downstream carotenoids.

## Conclusion

In summary, a multimodal Raman and MS imaging method has been successfully demonstrated in the current study to measure the spatial dependence of biochemical changes from gene silencing at the cellular level. This information is vital in gaining a complete understanding of the loss-of function phenotypes produced by VIGS. The most common analysis of VIGS efficacy provides an average percentage change in the mRNA levels with no spatial correlation of biochemical changes at the cellular level. We report in regions of the leaf that show a visible mosaic phenotype, the carotenoid expression is reduced but not completely eliminated. The presented multimodal imaging approach will be useful to study the silencing of plant genes that produce a unique biochemical signature even if the gene silencing does not produce a visible phenotype. If the phenotype is not visible, it is advisable to sample multiple locations, possibly multiple tissues, to ensure heterogeneous biochemical responses are measured. We have measured up to six locations on the same leaf (data not shown) without observing any signal degradation, and more measurements should be possible on the same leaf. This proof-of-concept study used the common VIGS marker gene, *pds*, which is easily detected by the characteristic variegated phenotype. We propose this method should be used in conjunction with gene expression analysis (e.g., qRT-PCR) to confirm that gene silencing was successful. The imaging approach can then be applied to measure resulting biochemical changes that result from the reduced gene expression. For example, the resulting biochemical changes may be measured for plant genes that alter the synthesis of lignin, cellulose, or carotenoids. Raman and MS imaging [56] can provide spatially-correlated biochemical compositions in the leaf, and stem of monocots (Additional file 1: Fig. S9) and dicots (Additional file 1: Fig. S10). In addition, the presented approach will also be useful for measuring biochemical changes that result, for example, from biotic and abiotic stresses.

## Additional file

**Additional file 1.** Additional materials and methods, images, and spectra.

## Abbreviations

*pds*: *phytoene desaturase*
FoMV: *Foxtail mosaic virus*
VIGS: virus-induced gene silencing
ORFs: open reading frames
GFP: green fluorescent protein
MS: mass spectrometry
MALDI: matrix-assisted laser desorption/ionization
HPLC: high performance liquid chromatography
