# Supplementary material for: Characterizing virus-induced gene silencing at the cellular level with in situ multimodal imaging
Source: Plant Methods. 2018 May 25;14:37. doi: 10.1186/s13007-018-0306-7 (PMC5968576; doi:10.1186/s13007-018-0306-7)
Supplement: Supplementary file 1 — Additional file 1. Additional materials and methods, images, and spectra. [file 13007_2018_306_MOESM1_ESM.docx]

**Characterizing Virus-Induced Gene Silencing at the Cellular Level with *In Situ* Multimodal Imaging**

Sadie J. Burkhow^a^, Nicole M. Stephens^a^, Yu Mei^b^, Maria Emilia Dueñas^a^, Daniel J. Freppon^a^, Geng Ding^c^, Shea C. Smith^d^, Young-Jin Lee^a^, Basil J. Nikolau^c^, Steven A. Whitham^b^, Emily A. Smith^a^*.

^a^The Ames Laboratory, U.S. Department of Energy, and Department of Chemistry, Iowa State University, Ames, IA 50011-3111, USA; ^b^Department of Plant Pathology and Microbiology, Iowa State University, Ames, Iowa 50011; ^c^Department of Biochemistry, Biophysics, and Molecular Biology, Center for Metabolic Biology, Iowa State University, Ames, IA 50011, USA; ^d^Engineering Research Center for Biorenewable Chemicals, Iowa State University, Ames, IA 50011, USA.

**Corresponding Author***

Emily A. Smith

Tel: 515-294-1424

Email: [esmith1@iastate.edu](mailto:esmith1@iastate.edu)

ORCID ID 0000-0001-7438-7808

Institutional Email Address of Authors: S.J.B. [sburkhow@iastate.edu](mailto:sburkhow@iastate.edu); N.M.S. [stephenm@iastate.edu](mailto:stephenm@iastate.edu); Y.M. [yumei@iastate.edu](mailto:yumei@iastate.edu); M.E.D. [mduenas@iastate.edu](mailto:mduenas@iastate.edu); D.J.F. [dfreppon@iastate.edu](mailto:dfreppon@iastate.edu); G. D. [gengding@iastate.edu](mailto:gengding@iastate.edu); S.C.S. [shea.christine22@gmail.com](mailto:shea.christine22@gmail.com); Y.J.L. [yjlee@iastate.edu](mailto:yjlee@iastate.edu); B.J.N. [dimmas@iastate.edu](mailto:dimmas@iastate.edu); S.A.W. swhitham@iastate.edu

**Materials and Methods**

*Carotenoid Extraction.* Sample preparation was modified from previously described methods [1, 2]. Leaf 5 of each maize plant was cut into ~100 mg aliquots at the half leaf length position and flash frozen in liquid nitrogen. Samples were pulverized to fine powders in liquid nitrogen using a mortar and pestle. A solution of 2-mL methanol/chloroform (5:4) containing 0.1% butylated hydroxytoluene (BHT, Sigma-Aldrich) and 20 µL of 1 mg/mL of canthaxanthin as an internal standard (Sigma-Aldrich) were added to the powder samples. Samples were incubated on ice for 10 minutes; 1.1 mL of 1 M NaCl (prepared with 0.1 M Tris-HCl buffer pH 7.5) was added to the samples and incubated on ice for another 10 minutes. The samples were centrifuged (1350 × *g*) for 10 minutes at room temperature. The organic fraction was collected, and the aqueous layer was centrifuged with an additional 1 mL aliquot of chloroform containing 0.1% BHT. The combined organic fractions were dried under a stream of nitrogen and the residue was dissolved in 200 µL ethyl acetate containing 0.1% BHT for future analysis.

An Agilent 1100 series high performance liquid chromatograph (HPLC) with photodiode array and mass selective (MS) detectors was used for carotenoid separation and identification. A Spherisorb® 5 μm ODS2 column (250×4.6 mm) (Phenomenex) heated to 40 ºC was utilized with a sample injection volume of 20 µL. The mobile phase consisted of 0.1% acetic acid (v:v) in acetonitrile/methanol/water (84:9:7, v:v:v; eluent A), and 0.1% acetic acid (v:v) in methanol/ethyl acetate (68:32, v:v; eluent B). The separation gradient with a flow rate of 1 mL/min was as follows: 0 min 0% B, 15 min 100% B, 28 min 100% B, 30 min 0% B, post run (5 min). A photodiode array detector was used for absorption-based detection. An atmospheric pressure chemical ionization source in positive mode was utilized with an ion trap mass spectrometer. The MS conditions were as follows: nebulizing nitrogen gas flow: 12 L/min; nebulizing gas pressure: 25 p.s.i.; dry temperature: 350 °C, vaporizer temperature: 400 °C; maximum accumulation time: 300 ms; mass scan range: 150 to 700 m/z; and corona: 3900 nA.

Quantification of all carotenoids were processed with DataAnalysis and QuantAnalysis software (Bruker Daltonics). Lutein (Sigma-Aldrich) and zeaxanthin (Sigma-Aldrich) were identified using commercial standards. Violaxanthin/neoxanthin, α-carotene, and β-carotene were identified by the m/z values of the molecular ions and by MS/MS analysis of the generated fragments. Retention times for carotenoids were as follows: violaxanthin/neoxanthin (8.1 min); lutein (9.9 min); zeaxanthin (10.2 min); canthaxanthin (10.9 min); α-carotene (19.9 min); and β-carotene (20.2 min). α and β-carotene could not be individually quantified due to overlapping peaks. The phytoene signal was too low to be measured in the FoMV and non-inoculated leaves with the utilized HPLC conditions and equipment. All reported uncertainties represent one standard deviation.

**Table**

| **Table S1. Average measured value (µg/g of fresh weight) by HPLC of the indicated carotenoids in maize leaf extract with uncertainty representing on standard deviation.^1^** | | | |
| --- | --- | --- | --- |
|  | FoMV-*pds^2^* | FoMV^3^ | Non-inoculated |
| α- & ß-carotene | 20 ± 3  (p = 0.5) | 11 ± 2  (p = 0.004) | 22 ± 1 |
| Phytoene | 0.4 ± 0.2 | 0.011 ± 0.005 | Not Detected^4^ |
| Violaxanthin | 8 ± 3  (p= 0.6) | 7 ± 2  (p= 0.2) | 10 ± 2 |
| Lutein | 54 ± 5  (p = 0.2) | 40 ± 10  (p = 0.2) | 61 ± 3 |
| Zeaxanthin | 2.4 ± 0.5  (p = 0.3) | 1 ± 1  (p= 0.3) | 7 ± 5 |

^1^ All measurements were collected from one leaf with 2 to 3 tissue replicates.

^2^ p-values comparing FoMV-*pds* vs. Non-Inoculated.

^3^ p-values comparing FoMV vs. Non-Inoculated.

^4^ The phytoene detection limit for this analysis is 0.003 µg/g of fresh weight

**Figures**


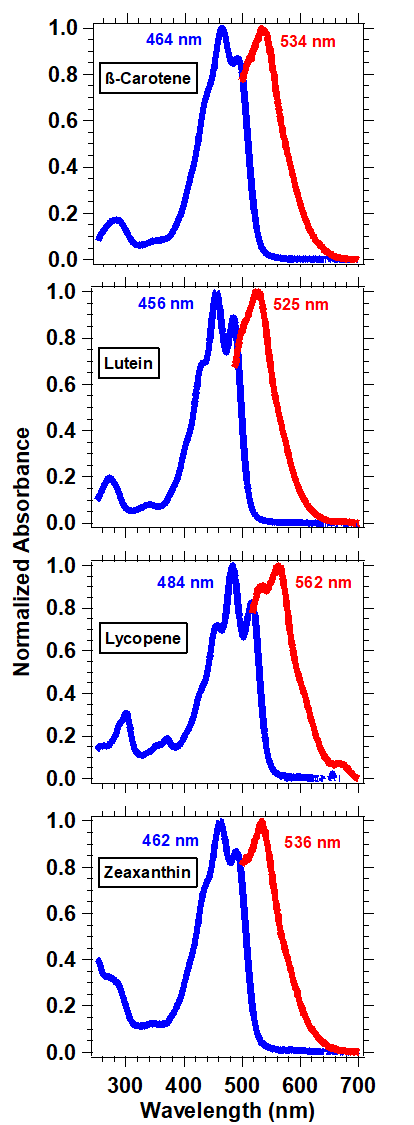


**Fig. S1.** Absorbance (blue) and fluorescence (red) spectra for ß-carotene, lutein, lycopene, and zeaxanthin dietary supplements. All supplements were diluted to 0.01 mg/mL in chloroform. Absorbance measurements were performed on an Agilent 8453 UV-visible spectrophotometer. Fluorescence measurements were carried out on an Agilent Cary Eclipse spectrophotometer. The corresponding wavelength of maximum absorbance and fluorescence is listed in each spectrum. The wavelength of maximum absorbance (fluorescence) was used as the excitation (emission) wavelength to collect the fluorescence (excitation) spectrum.


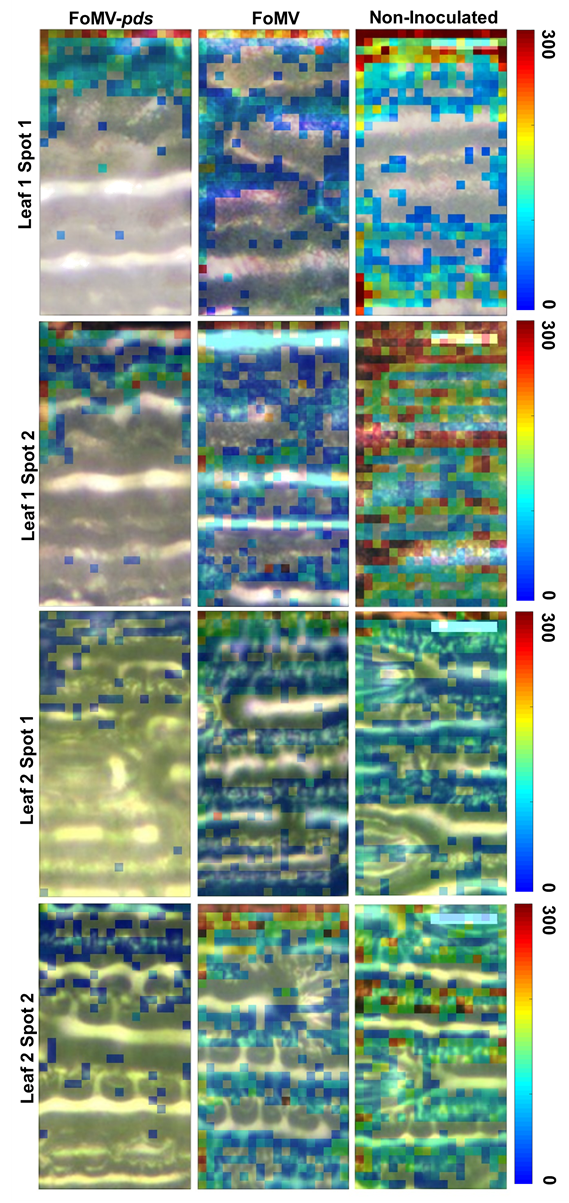


**Fig. S2.** Merged optical and ~1520 cm^-1^ Raman images (color scale) of *in situ* whole maize leaves. All maps were scaled to the same intensities. The scale bar is 25 µm. Optical images of an expanded area around where the Raman images were collected is seen in Supplemental Fig. S3.

**
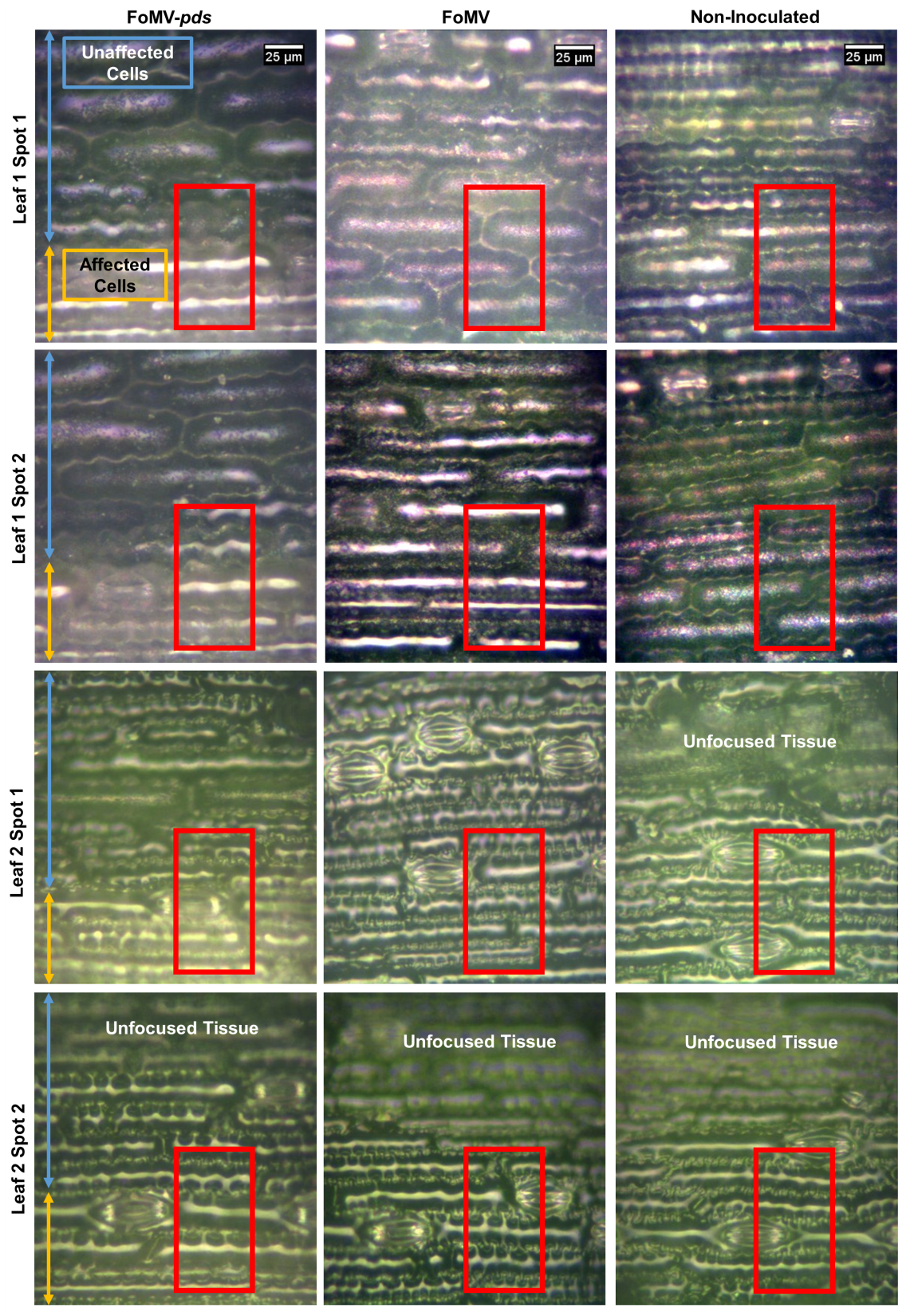
**

**Fig. S3.** Optical images showing the location (red box) where the *in situ* whole leaf measurements presented in Figure 3 and Supplemental Fig. S2 were collected. Within the FoMV-*pds* optical images, the variegated white phenotype is shown with a yellow arrow and the unaffected area is shown with a blue arrow. The leaf is not optically flat, and some regions are out of focus (as noted).


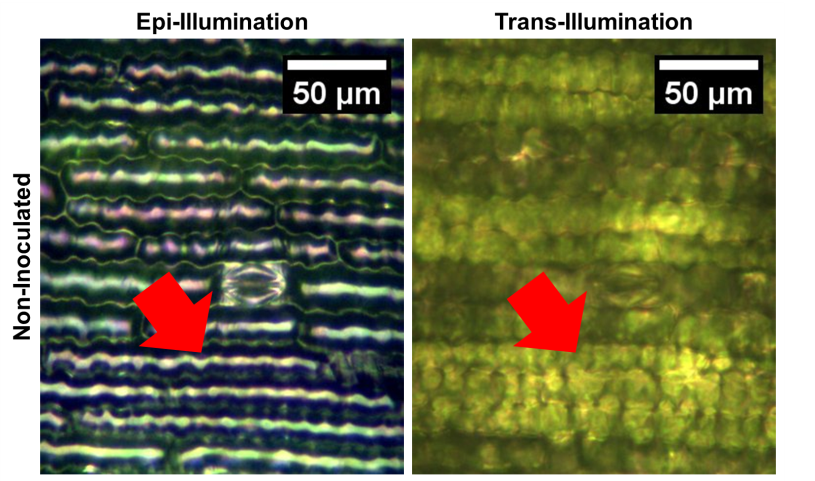


**Fig. S4.** White light optical images of a non-inoculated leaf with epi-illumination (incident light and reflected light on the same side of the sample) and trans-illumination (incident light and transmitted light on opposite sides of the sample). The red arrow highlights the same location of the leaf in both images.

**
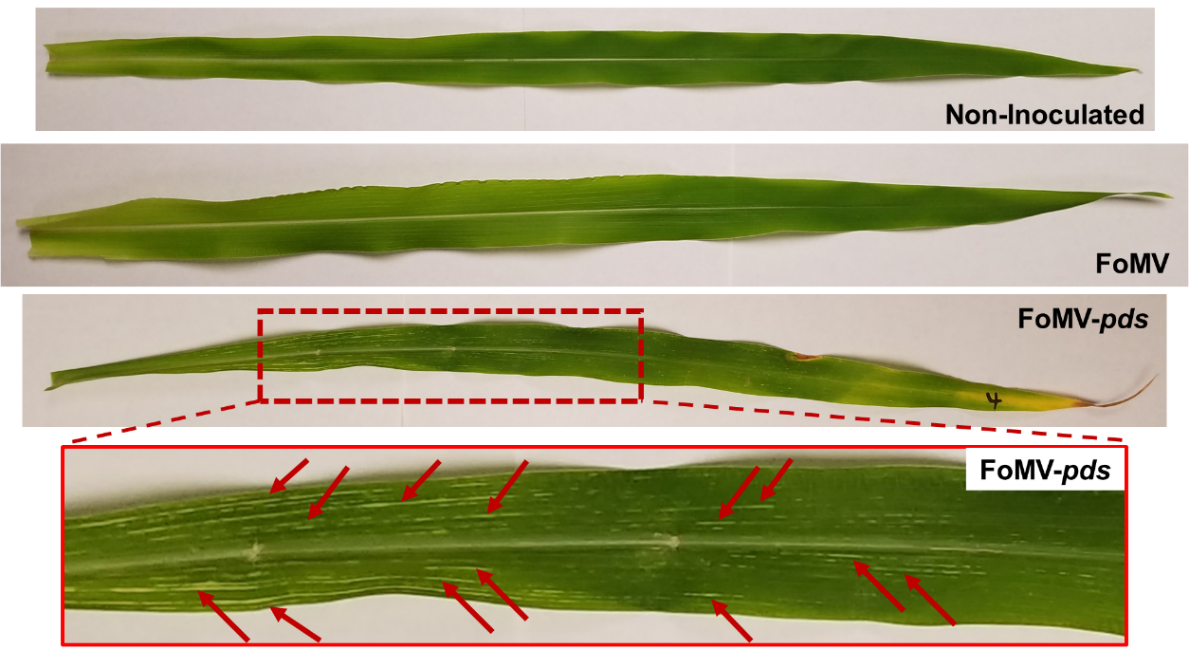
**

**Fig. S5.** Representative optical images of the entire leaf of the three plant types. The total leaf length ranged between 12-18 inches. Within the FoMV-*pds* magnified optical image (bottom image), example variegated areas are highlighted with red arrows. The percentage of the FoMV-*pds* leaf exhibiting the variegated area within the lower optical image was 13%. This was measured using ImageJ and by applying a color threshold to identify the areas lacking green coloration. The total white area identified by the color threshold was divided by the total leaf area. The variegated areas of the FoMV-*pds* leaf do not provide information about the biochemical composition or microscopic information, information that is measured with the presented imaging approach.

**
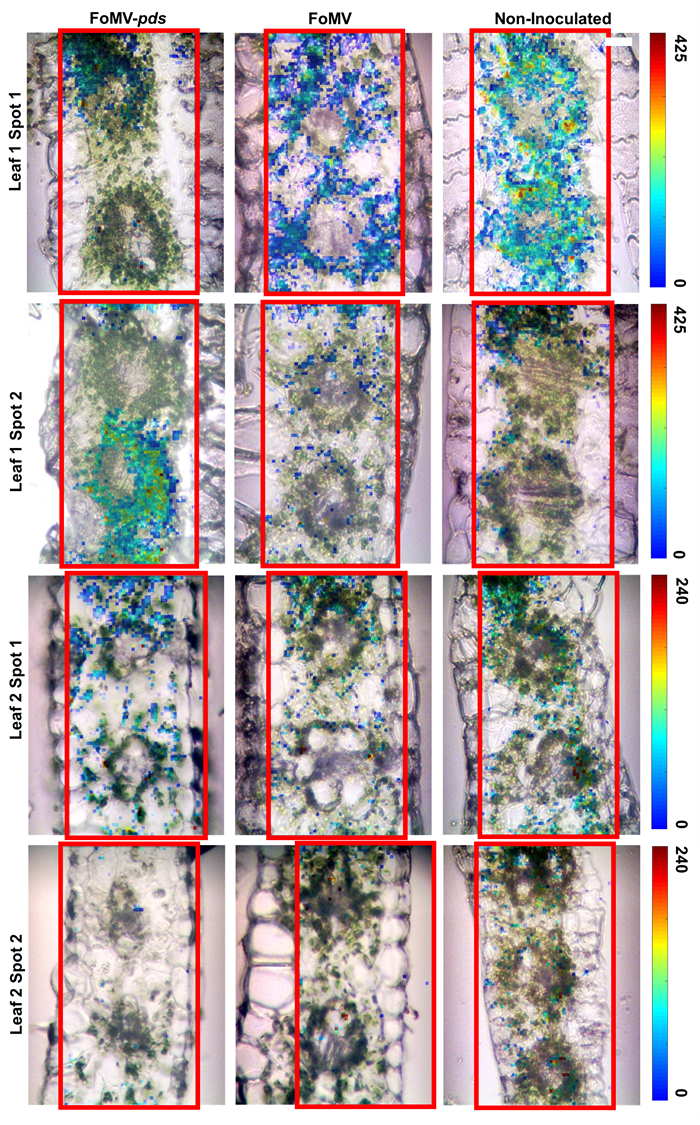
**

**Fig. S6.** Merged optical and ~1520 cm^-1^ Raman images (color scale and outlined in red) of maize leaf transverse cross sections. Scale bar is 25 µm.


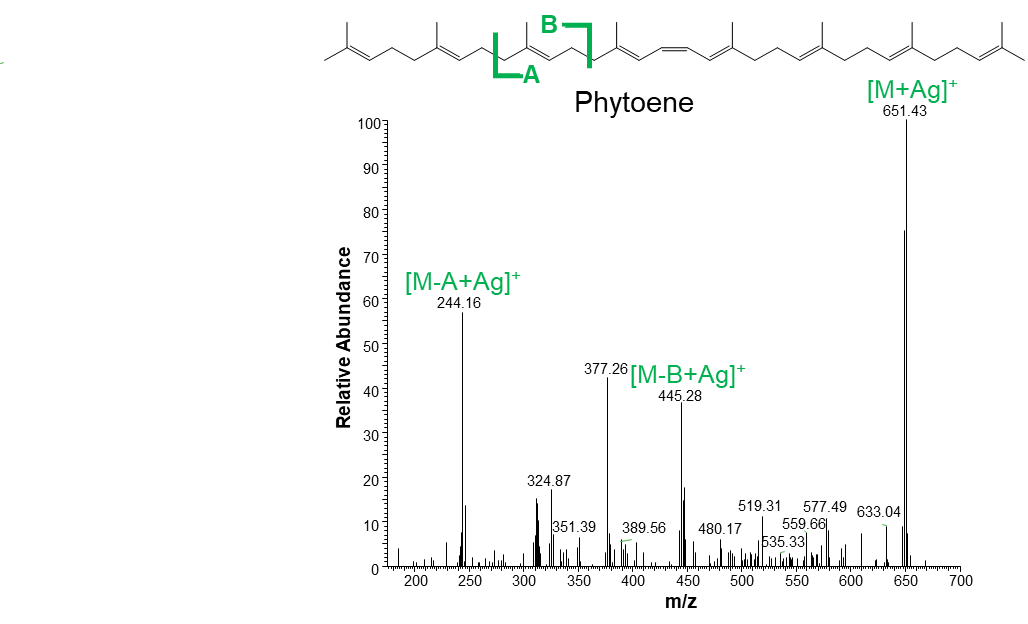


**Fig. S7.** Tandem (MS/MS) mass spectrum of phytoene, (*m/z* 651.405, [M+^107^Ag]^+^) from FoMV-*pds* fractured leaves.


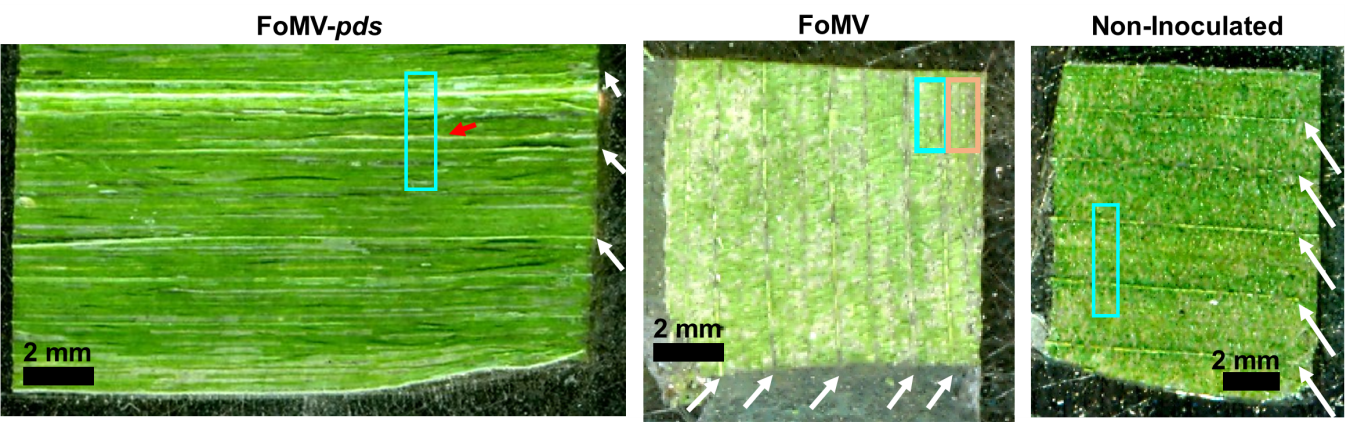


**Fig. S8.** Larger optical images showing the locations (light blue & orange boxes) where the fractured leaf MS imaging measurements presented in Fig. 6 were collected. Within the FoMV optical image, the orange box indicates the top MS image within Fig. 6, and the light blue represents the bottom MS image. The leaves are orientated in the same direction as shown in Fig. 6. The leaf veins within all three plant types are outlined with white arrows. Within the FoMV-*pds* optical image, an example variegated white area is highlighted with a red arrow. Note these leaf fractures were less than 2 inches in length; sample optical images of an entire leaf are shown in Supplemental Fig. S5.


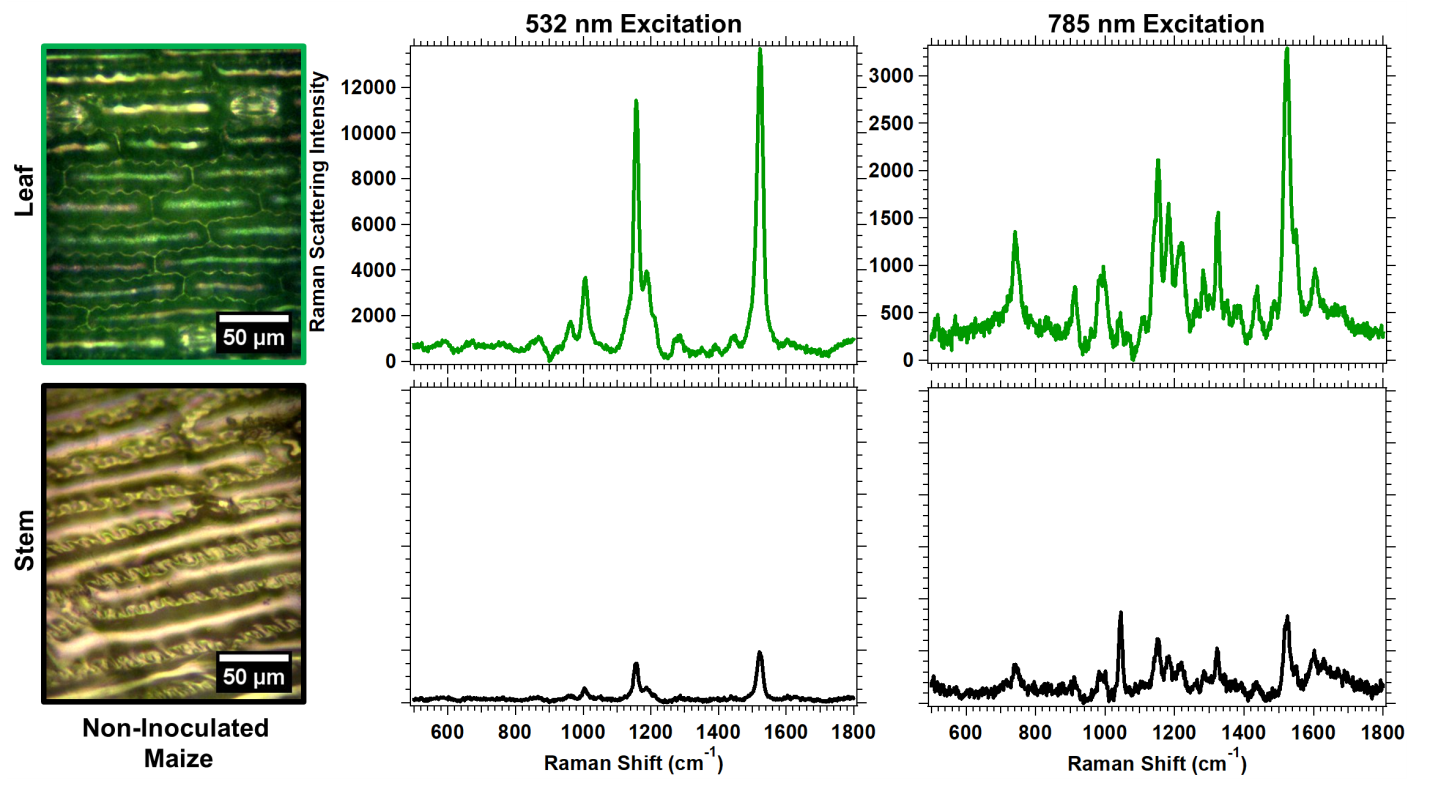


**Fig. S9.** Maize (monocot) leaf and stem tissues. (Left) optical image of selected regions of the indicated tissue and background subtracted Raman spectra collected with (middle) 532 nm and (right) 785 nm excitation. Using 532 nm excitation, the Raman spectrum is dominated by carotenoid peaks (~1520, ~1150, and ~1000 cm^-1^). Using 785 nm excitation, the Raman signal includes the polysaccharide, lignin (e.g., the lignin peak at ~1605 cm^-1^), and other biomolecule peaks [3].


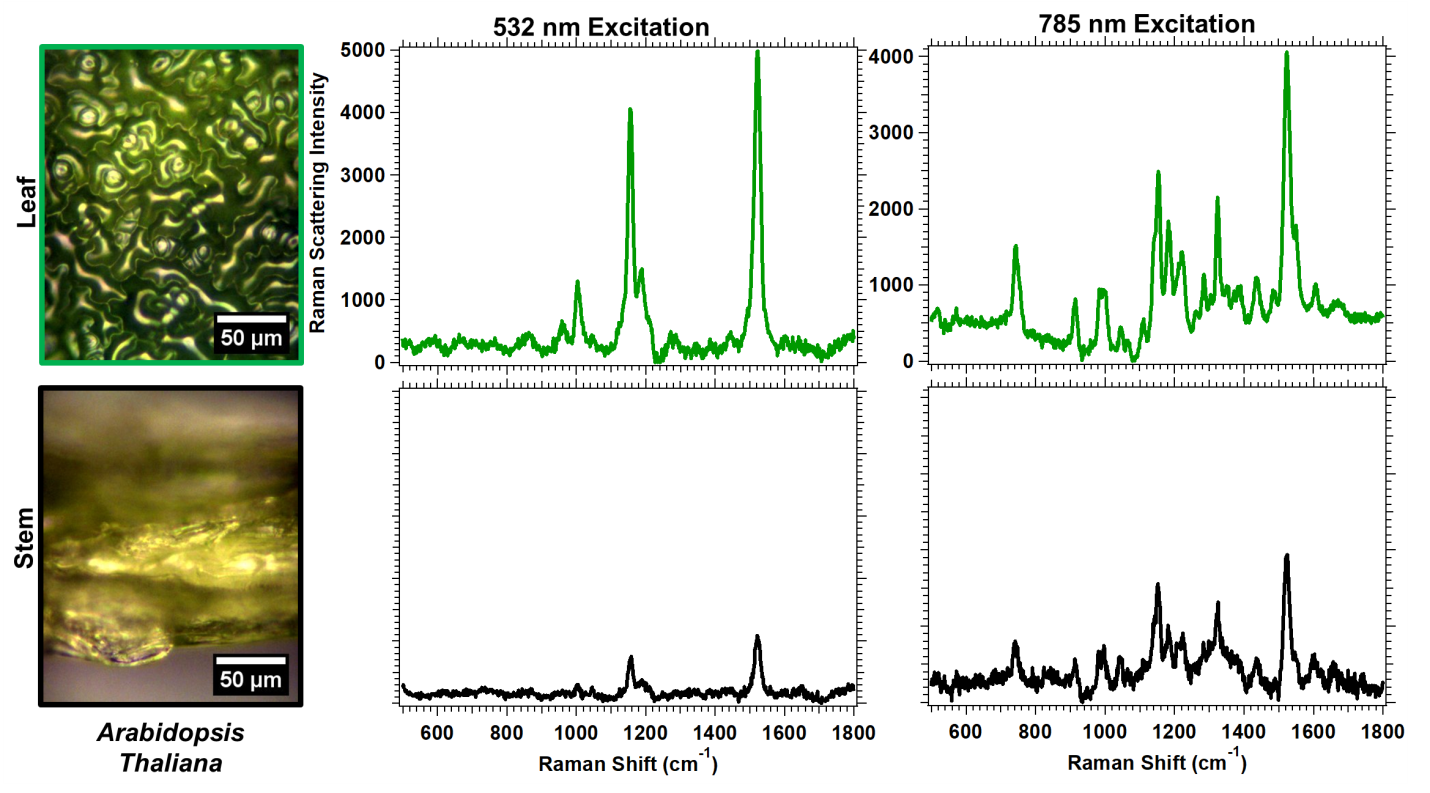


**Fig. S10.** *Arabidopsis thaliana* (dicot) leaf and stem tissues. (Left) optical image of selected regions of the indicated tissue and background subtracted Raman spectra collected with (middle) 532 nm and (right) 785 nm excitation. Using 532 nm excitation, the Raman spectrum is dominated by carotenoid peaks (~1520, ~1150, and ~1000 cm^-1^). Using 785 nm excitation, the Raman signal includes polysaccharides (e.g., the ~1043 cm^-1^ peak commonly assigned to cellulose that is prominent in the leaf spectrum), lignin, and other biomolecule peaks [3].

References

1. Bino RJ, De Vos CHR, Lieberman M, Hall RD, Bovy A, Jonker HH, Tikunov Y, Lommen A, Moco S, Levin I. The light-hyperresponsive high pigment-2dg mutation of tomato: alterations in the fruit metabolome. New Phytologist. 2005;166:427-438.

2. López-Ráez JA, Charnikhova T, Gómez-Roldán V, Matusova R, Kohlen W, De Vos R, Verstappen F, Puech-Pages V, Bécard G, Mulder P *et al*. Tomato strigolactones are derived from carotenoids and their biosynthesis is promoted by phosphate starvation. New Phytologist. 2008;178:863-874.

3. Lupoi JS, Gjersing E, Davis MF. Evaluating Lignocellulosic Biomass, Its Derivatives, and Downstream Products with Raman Spectroscopy. Frontiers in Bioengineering and Biotechnology. 2015;3:50.
